# Antipsychotic prescribing for vulnerable populations: a clinical audit at an acute Australian mental health unit at two-time points

**DOI:** 10.1186/s12888-017-1295-1

**Published:** 2017-04-13

**Authors:** Sara S McMillan, Sara Jacobs, Louise Wilson, Theo Theodoros, Gail Robinson, Claire Anderson, Gabor Mihala, Amanda J Wheeler

**Affiliations:** 1 Menzies Health Insitute, Griffith University, Brisbane, Australia; 2 Division of Pharmacy Practice and Policy, School of Pharmacy, University of Nottingham, Nottingham, UK; 3 Faculty of Medicine, University of Queensland, Brisbane, Australia; 4 Royal Australia and New Zealand College of Psychiatrists Trainee, Melbourne, Victoria Australia; 5 Metro South Addiction and Mental Health Services, Department of Health, Brisbane, Queensland Australia; 6 Metro North Mental Health Services, Department of Health, Brisbane, Queensland Australia; 7 Faculty of Medical and Health Sciences, Auckland University, Auckland, New Zealand

**Keywords:** Audit, Antipsychotics, Mental Illness

## Abstract

**Background:**

Antipsychotics are recognised as a critical intervention for schizophrenia and bipolar disorder. Guidelines globally endorse the routine practice of antipsychotic monotherapy, at the minimum effective dose. Even in treatment-resistant schizophrenia, clozapine use is endorsed before combining antipsychotics.

This aim of this study was to review antipsychotic polytherapy alone, high-dose therapy alone, polytherapy and high-dose prescribing patterns in adults discharged from an inpatient mental health unit at two time-points, and the alignment of this prescribing with clinical guideline recommendations. Additionally, associations with polytherapy and high-dose antipsychotic prescribing, including patient and clinical characteristics, were explored.

**Methods:**

A retrospective clinical audit of 400 adults (200 patients at two different time-points) discharged with at least one antipsychotic. Preliminary findings and education sessions were provided to physicians between Cohorts. Outcomes (polytherapy alone, high-dose therapy alone, polytherapy and high-dose therapy) were compared between study Cohorts using chi-squared and rank-sum tests. Associations between outcomes and covariates were assessed using multivariable logistic regression.

**Results:**

Most patients (62.5%) were discharged on a single antipsychotic within the recommended dose range. There was a clear preference for prescribing second generation antipsychotics, and in this respect, prescribing is aligned with current evidence-based guidelines. However, sub-optimal prescribing practices were identified for both Cohorts in relation to polytherapy and high-dose antipsychotic rates. Involuntary treatment, frequent hospitalisations and previous clozapine use significantly increased the risk of all three prescribing outcomes at discharge.

**Conclusions:**

In a significant minority, antipsychotic prescribing did not align with clinical guidelines despite increased training, indicating that the education program alone was ineffective at positively influencing antipsychotic prescribing practices. Further consideration should be given when prescribing antipsychotics for involuntary patients, people with frequent hospitalisations, and those who have previously trialled clozapine.

## Background

Optimal management of serious mental illnesses, such as schizophrenia, bipolar affective disorder and other psychotic disorders, focuses on both symptom and functional recovery with a range of pharmacological, psychosocial and psychological interventions. Antipsychotic medication is widely recognised as a critical intervention in both acute and ongoing treatment of schizophrenia [1, 2], and more recently in bipolar disorder [3, 4].

Clinical practice guidelines globally endorse the routine practice of monotherapy with antipsychotic medication, at the minimum effective dose, for people with schizophrenia or bipolar disorder [1, 2, 4, 5]. Even in treatment-resistant schizophrenia, i.e. treatment failure with two different antipsychotic agents, clozapine use is endorsed before combining antipsychotic therapies [1, 2, 5, 6]. The use of more than one antipsychotic is only recommended for short periods when switching treatments [7], or for people with treatment-resistant schizophrenia who have had only a partial response to clozapine [1]. Other plausible reasons include: if a person refuses to take clozapine, or there is a contraindication or intolerability to clozapine as it is associated with a significant side effect profile [8]. Reasons for clozapine ineffectiveness must be explored before adding a second antipsychotic, e.g. consumer engagement with clozapine therapy (i.e. adherence) and other medication use [1]. In their review of treatment-resistant schizophrenia, Dold and Leucht confirmed the continuing uncertainty as to which antipsychotic to recommend if clozapine is inappropriate [9]. Current Australian guidelines outline two choices: i) augmentation with another antipsychotic or electroconvulsive therapy, or ii) switch to another agent [2].

In spite of the above recommendations, antipsychotic polytherapy, or high-dose antipsychotic prescribing, or both, appears to be common practice [10–12]. Between 1970 and 2009, the globally pooled median antipsychotic polytherapy rate was 19.6% [13]. The 2010 Australian National Survey of Psychosis identified that 28% (*n* = 240) of participants with schizophrenia were taking two or more antipsychotics [14]. There are significant concerns with antipsychotic polytherapy and dosing above therapeutic recommendations. These include, but are not limited to, lack of evidence to support efficacy [15], reduced medication adherence, drug interactions, increased treatment burden, e.g. financial costs and adverse effects, and mortality [7, 16].

Variables suggested to be associated with antipsychotic polytherapy include residual psychotic symptoms [17], being treated involuntarily [18], psychiatrists prescribing beliefs and clinical experience [19], and hospitalisation or longer hospital stays, which may be influenced by duration of illness [20–23]. One of the strongest predictors of high antipsychotic doses is the prescribing of more than one antipsychotic concurrently [23–26]. Overall, there is conflicting evidence for associations between patient characteristics and either antipsychotic polytherapy or high-dosing, such as age [18, 23, 27, 28] and ethnicity [27–31]. For instance males have been reported to be at greater risk, or have higher rates of these outcomes [18, 23, 27, 29], yet this was not demonstrated in New Zealand [28].

Audit and feedback (clinical audit) is an intervention commonly undertaken by organisations to monitor and improve the quality of health care. A clinical audit ‘*compares actual practice with an optimal standard of practice*’ [32]; this is integral for evaluating and improving practices and ultimately, patient safety and care. It is a cycle that follows a systematic process of establishing best practice, evaluating care against explicit criteria, implementing steps to improve care, and monitoring for continual improvement [33, 34]. The aim of this paper is to review polytherapy and high-dose antipsychotic prescribing patterns in adults discharged from an inpatient mental health unit at two time-points, and the alignment of this prescribing with clinical guideline recommendations. Additionally, this paper explores associations with polytherapy and high-dose antipsychotic prescribing, including patient and clinical characteristics.

## Methods

This study was a single-centre, retrospective clinical audit of 400 adults discharged with a prescription for at least one antipsychotic medication from a Queensland-based metropolitan public hospital. The hospital is the largest of three adult psychiatric hospitals in the region covering a catchment area of approximately one million people. The hospital functions as a major teaching hospital, and has 67 adult psychiatric inpatient beds, which treats both voluntary and involuntary patients admitted under the relevant legislation [35]. The catchment area also encompasses a large culturally and linguistically diverse population.

Information was obtained for 200 patients at two different time-points: patients discharged on or before the 31st January 2014 (Cohort 1), and on or before the 31st January 2015 (Cohort 2). This sample size was deemed large enough to provide adequate statistical power and an accurate reflection of hospital prescribing practices.

The time points were chosen to allow for education interventions to be provided at the start of a new trainee year, with trainees beginning their first term of 2014 at the start of February. Rotations for training psychiatry registrars through the Royal Australian and New Zealand College of Psychiatrists (RANZCP) are generally 6 months’ duration for full-time trainees [36]. It was anticipated that education interventions would occur over a period of 5–10 months. It was acknowledged that the majority of education interventions were targeted towards training psychiatrists. This was done in a purposeful manner with training psychiatrists noted to generally have greater contact with patients and more frequent review of psychotropic medication prescription (under the guidance of supervising psychiatrists).

At the time of study (between Cohorts), the hospital ran a regular education program on Monday afternoons, for mental health medical staff (psychiatrists and training psychiatrists). Antipsychotic education was included during these sessions in a number of ways such as: a one-hour presentation by a pharmacy student highlighting the issues from a preliminary analysis of Cohort 1 findings; a one-hour presentation that was video-conferenced to three hospital sites across the district presented by a training psychiatrist which specifically addressed high-dose antipsychotic prescribing and antipsychotic polypharmacy. In addition, four pharmacology sessions were provided to psychiatric trainees discussing the rational and appropriate use of psychotropic medications. These teaching sessions were conducted from February to October 2014. Pharmacology teaching to training psychiatrists was positively received, whilst education sessions on antipsychotic polypharmacy and high-dose antipsychotic use presented to consultant psychiatrists and training psychiatrists were met with a variety of responses, including defence of antipsychotic polypharmacy despite a lack of supporting evidence. Hospital mental health pharmacists were also invited to attend these education sessions, and a dedicated presentation of findings from Cohort 1 was presented to the greater hospital pharmacy group.

Ethical approval was obtained from both a University (HSV/04/15/HREC) and District Health Board (EC00167) Ethics Committee. The records for the most recent 200 adult patients discharged prior to each of the two time-points with at least one antipsychotic prescribed, irrespective of diagnosis, were obtained (Cohort 1: January 31, 2014 to November 27, 2013; Cohort 2: January 31, 2015 to December 2, 2014). Patients were excluded if they were transferred to another ward, unit or hospital, became ‘absent without permission’ during their inpatient admission, or died during the admission (82 and 73 patients were excluded from each cohort respectively). For patients with multiple hospital discharges, only the most recent discharge in each Cohort was included (i.e. the one closest to the 31st January for each time-point). Nineteen patients were common to both cohorts. There were no significant changes made to the service delivery model between the two review periods.

**Fig. 1:**
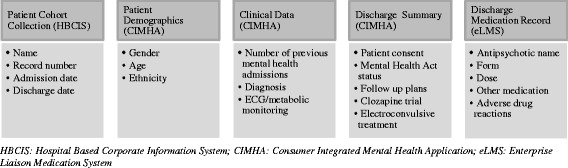
Data collection process

Three electronic databases were used, by two psychiatry registrars (Cohort 1) and two Master of Pharmacy students (Cohort 2) to obtain study data (Fig. 1). Information was de-identified with a unique identifier used for study data entry in Microsoft Excel^®^. A briefing session and procedure guide ensured that all researchers followed a standardised data collection process. A post-data entry meeting was held to combine data from the two Cohorts, and to identify and resolve any discrepancies. The following definitions were used for data collection and analysis by the researchers:

- Antipsychotic polytherapy: prescribing of two or more different antipsychotic agents simultaneously, or prescribing two or more different formulations of the same antipsychotic medication, including ‘when required;’
- High-dose prescribing (total antipsychotic equivalent dose): The sum of the total daily dose (TDD) of each antipsychotic used by a patient within 24 h divided by the recommended maximum daily dose [37, 38]. A total equivalent dose greater than one was classified as a high-dose;
- Ethnicity: country of birth; and,
- Involuntary treatment: included both an Involuntary Treatment Order (ITO) and a Forensic Order (FO). An ITO is endorsed by a psychiatrist and authorises a person to receive mental health treatment without consent. Alternatively, an FO is endorsed by the legal system [35].

### Analysis

Data was imported into Stata 14.1 for cleaning and analysis. Results were presented as frequencies (n) and proportions (%) for categorical data, or median and 25th/75th percentiles for continuous non-symmetrical data. Baseline characteristics were compared between cohorts using chi-squared and rank-sum tests. Associations between therapy received (polytherapy alone, high-dose therapy alone, poly- and high-dose therapies) and cohort and other covariates were assessed with odds ratios, which were calculated using multivariable logistic regression. Correlations between covariates were checked using linear regression or by calculating pairwise correlation coefficients (all absolute values *r* < 0.40). Three separate multivariable models (dependent variable: therapy received) were built using covariates presented in Table 4 including cohort. The models were derived through manual backwards step-wise removal of covariates at *p* ≥ 0.05 level. Statistical significance was declared at *p* < 0.05. Only significant odds ratios were presented. Missing values were not imputed. No adjustment was made for multiple comparisons.

## Results

Overall, the majority of discharged patients in both Cohorts were male, with a similar median age (38 years, Cohort 1; 35 years Cohort 2; Table 1). Over three-quarters of Cohort 1 (*n* = 152; 76.0%) and two-thirds of Cohort 2 (*n* = 137; 68.0%) participants were Australian born. Similar proportions were identified between the two Cohorts with respect to primary diagnosis; the most prominent condition overall in the study population was schizophrenia (*n* = 148; 37.0%). The only significant difference identified between Cohorts was the higher rate of comorbid substance use recorded for Cohort 1 (*p* = 0.001).

**Table 1:** Participant characteristics by Cohorts (*n* = 400)

	Cohort 1 *n* = 200	Cohort 2 *n* = 200	Total *n* = 400	*p*-value
Group size	200 (50%)	200 (50%)	400 (100%)	
Gender (male)	117 (58%)	119 (60%)	236 (59%)	0.839
Age (years)^a^	38 (28–48)	35 (28–44)	36 (28–46)	0.059^b^
Ethnicity				
*- Australian*	152 (76%)	137 (68%)	289 (72%)	0.056
*- Asian*	20 (10%)	14 (7%)	34 (8%)	
*- Pacific Islander*	13 (6%)	23 (12%)	36 (9%)	
*- Other*	15 (8%)	26 (13%)	41 (10%)	
Admissions (last 12 months)				
*- One or two*	157 (78%)	165 (82%)	322 (80%)	0.582
*- Three or four*	27 (14%)	21 (10%)	48 (12%)	
*- Five or more*	16 (8%)	14 (7%)	30 (8%)	
Hospital duration (days)^a^	8 (4–17)	10 (5–22)	9 (5–19)	0.317^c^
Primary diagnosis				
*- Schizophrenia*	72 (36%)	76 (38%)	148 (37%)	0.946
*- Bipolar disorder*	27 (14%)	26 (13%)	53 (13%)	
*- Other psychotic disorder*	30 (15%)	32 (16%)	62 (16%)	
*- Other*	71 (36%)	66 (33%)	137 (34%)	
Comorbid substance use disorder	77 (38%)	46 (23%)	123 (31%)	0.001
Involuntary treatment	77 (38%)	84 (42%)	161 (40%)	0.475
Previous clozapine trial	23 (12%)	20 (10%)	43 (11%)	0.628

n (%) shown unless otherwise noted

^a^median and inter-quartile range (25th and 75th percentiles) shown; *p*-values calculated with chi-squared test unless otherwise noted

^b^Wilcoxon rank-sum test

^c^median test

Prescribing practices across the two Cohorts were similar in terms of the antipsychotic medication used (Table 2). Second generation antipsychotics (SGA) were the most commonly prescribed antipsychotics, in particular risperidone was the most commonly prescribed agent (oral (PO) and long-acting injection (LAI), *n* = 125), followed by olanzapine (PO and LAI, *n* = 123), quetiapine (PO only, *n* = 85) and paliperidone (PO and LAI, *n* = 64).

**Table 2:** Antipsychotics prescribed on discharge

Class	Route	First AP		Second AP		Third AP	
SGA		C1	C2	C1	C2	C1	C2
Amisulpride	PO	2	5	2	5		
Aripiprazole	LAI		1				
	PO	4	10	2	1		
Asenapine	PO	2			1		
Clozapine	PO	17	13		2		
Olanzapine	LAI	4			1		
	PO	45	45	12	16		
Paliperidone	LAI	18	34	3	7		
	PO		1	1			
Quetiapine	PO	39	31	6	7	2	
Rispiridone	LAI	13	6	5	4		
	PO	34	38	11	14		
Ziprasidone	PO	3	1				
Subtotal	IM	35	41	8	12		
	PO	146	144	34	46	2	
FGA		C1	C2	C1	C2	C1	C2
Chlorpromazine	PO	2	2	1	3	2	
Flupenthixol	LAI	2	2	3	3		
Fluphenazine	LAI				2		
Haloperidol	LAI	2		1			
	PO	2	1	1	1		
Zuclopenthixol	LAI	11	10	5	6		
	PO			2	1		
*Subtotal*	IM	15	12	9	11		
	PO	4	3	4	5	2	
*AP Total*	IM	50	53	17	23		
	PO	150	147	38	51	4	
TOTAL		200	200	55	74	4	

*SGA* Second generation antipsychotic, *FGA* First generation antipsychotic, *PO* oral*; LAI* Long-acting injection*,* AP Antipsychotic*, C1* Cohort 1*, C2* Cohort 2

Table 3 shows that the majority of patients (*n* = 250/400; 62.5%) were discharged on a single antipsychotic medication with a dose within the therapeutic recommendations, i.e. below the maximum recommended dose (*n* = 132/200, 66.0% Cohort 1; *n* = 118/200, 59.0% Cohort 2). Overall antipsychotic polytherapy as a prescribing outcome at discharge (either alone or with high-dose antipsychotic therapy) was found in just less than a third (*n* = 130/400; 32.5%) of the study population (*n* = 56, 28.0% Cohort 1; *n* = 74, 37.0% Cohort 2). Overall high-dose antipsychotic therapy as a prescribing outcome at discharge (either alone or with antipsychotic polytherapy) was found in 20.5% (*n* = 82/400) of the study population (*n* = 36/200, 18.0% Cohort 1; *n* = 46/200, 23.0% Cohort 2). A total of 62 patients (15.5%) were prescribed both antipsychotic polytherapy and high-dose therapy at discharge (*n* = 24/200, 12.0% Cohort 1; *n* = 38/200, 19.0% Cohort 2).

**Table 3:** Therapy received by cohort

Therapy received		Frequency		
Polytherapy	High dose	Cohort 1 *n* = 200	Cohort 2 *n* = 200	Total *N* = 400
No	no	132	118	250
Yes	no	32	36	68
No	yes	12	8	20
Yes	yes	24	38	62

### Associations with polytherapy and high-dose antipsychotic prescribing

Associations between three antipsychotic prescribing outcomes at discharge: (i) polytherapy only; (ii) high-dose therapy only; and (iii) polytherapy and high-dose therapy together were investigated in a regression model with the following variables: Cohort, participant demographics, hospital admissions in the last 12 months, primary diagnosis, comorbid substance use disorder, involuntary treatment status and previous clozapine trial (Table 4).

**Table 4:** Multivariable logistic regressions (associations between therapy received and cohort/other covariates)

	Adjusted odds ratio (95% CI) of therapy received		
Therapy received	Polytherapy	High dose	Polytherapy and high dose
Model size	*n =* 400	*n =* 400	*n =* 312
Cohort (base: 1st)	1.81 (1.08–3.02)*	-	2.14 (1.10–4.17)*
Gender (base: male)	-	-	-
Age (year)	-	-	-
Ethnicity (base: Australian)	-	-	-
Adm. in last 12 m (base: one or two):			
- three or four	1.77 (0.87–3.60)	-	2.28 (0.97–5.35)
- five or more	7.52 (2.92–19.4)*	-	5.30 (1.57–17.8)*
Hospital stay (day)	^	^	^
Primary diagn. (base: schizophrenia):			
- bipolar disorder	0.19 (0.08–0.47)*	-	0.26 (0.08–0.80)*
- other psychotic disorder	0.33 (0.14–0.75)*	-	0.33 (0.11–1.00)
- other	0.40 (0.21–0.76)*	-	0.40 (0.17–0.91)*
Comorbid substance use disorder	-	-	-
Involuntary treatment	3.95 (2.31–6.77)*	2.22 (1.34–3.66)*	3.18 (1.61–6.29)*
Previous clozapine trial	5.80 (2.43–13.8)*	2.19 (1.10–4.38)*	5.58 (2.04–15.3)*

*CI* confidence interval, * *p* < 0.05; hyphen = excluded from multivariable model at *p* ≥ 0.50; ^ = excluded due to multicorreality; *m* months, *diagn.* diagnosis, *adm.* admissions

There was a difference between Cohorts with respect to two outcomes: (i) polytherapy alone, and (iii) polytherapy and high-dose together; patients in Cohort 2 were 1.8 times more likely to be discharged on polytherapy alone and more than twice as likely to be discharged on polytherapy and high-dose therapy together. No significant associations were identified between the three outcomes and patients’ age, gender, ethnicity, and having a comorbid substance use disorder.

Those patients who had five or more admissions in the previous 12 months were significantly more likely to be discharged on (i) antipsychotic poytherapy alone and (iii) polytherapy and high-dose therapy together; the risk of polytherapy alone at discharge was 7.5 times more likely and polytherapy and high-dose therapy together was 5.3 times more likely compared to those patients admitted less frequently.

Compared to patients with schizophrenia, those with all other diagnoses were significantly less likely to be prescribed polytherapy alone. The likelihood of antipsychotic polytherapy plus high-dose therapy was similar between patients diagnosed with schizophrenia and those with other psychotic disorders, but was significantly lower for those with bipolar and other disorders.

Involuntary treatment and previous clozapine use significantly increased the risk of all three prescribing outcomes at discharge (Table 4).

## Discussion

Overall, this study found that the majority of patients (62.5%) were discharged on a single antipsychotic within the recommended dose range. There was a clear preference for prescribing SGAs, which are recommended first-line agents for schizophrenia and schizoaffective disorder by the RANZCP [2]. In this respect, prescribing is aligned with current evidence-based guidelines. However, sub-optimal prescribing practices were identified for *both* Cohorts in relation to polytherapy and high-dose antipsychotic rates. Subsequently, a number of patients were at risk of adverse consequences as a result of their discharge treatment. This was despite increased clinician education prior to the second time-point (Cohort 2), reflecting the need for a review of the training provided at a local level and in wider medical education. Our audit results provide further evidence that gender, age and ethnicity are not associated with polytherapy or high dose [26, 28], although the ethnic minority groups made up only about 30% of each Cohort. Patients with a primary diagnosis of schizophrenia were identified to be at significantly greater risk of polytherapy alone or polytherapy and high dose antipsychotic use. Lastly, polytherapy alone, high antipsychotic dose alone, and polytherapy/high-dose therapy combined were significantly associated with the following three consumer variables: five or more previous hospital admissions within the last 12 months, involuntary treatment and previous clozapine use.

These results should be considered with respect to other recent research in this area. A similar audit was conducted with 272 inpatients in Portugal in 2012 [39]. Compared to our study, Campose-Mendes et al. identified a higher proportion of patients on polytherapy, as well as those on high dose therapy [39]; this may be expected with an audit undertaken in 12 psychiatric units compared to our one. Involuntary treatment and prior psychiatric admission was found to be associated with high dose in both studies [39]. Our study did not explore associations depending on the formulation of the antipsychotic or employment status.

The frequency of antipsychotic polytherapy alone was similar between the two Cohorts, and it was encouraging to see that the majority of patients were being treated with one antipsychotic. The total rates of any antipsychotic polytherapy in this study were lower than those reported in a small Australian audit of hospital inpatients with schizophrenia [40]; Malhi et al. did not investigate associated high-dose antipsychotic prescribing.

While our relatively low rate of antipsychotic polytherapy alone could be viewed positively, this needs to be considered in association with high dosing or whether clozapine has been trialled. A similar proportion of patients were discharged with polytherapy and high-dose therapy, particularly those patients in Cohort 2. An investigation into *what* drives this outcome is needed in this clinical setting; was a second (or third) antipsychotic agent added after an increased dose or vice versa? As polytherapy and high antipsychotic dosing escalates the risk of adverse effects, which can lead to rehospitalisation, the consequences of, and lack of supportive evidence for such a combination, must be reiterated to clinicians. This audit emphasises the need to develop further strategies to reduce these rates in this particular hospital. However, it is noted that the audit specifically gathered clinical information from discharge summaries and pharmacy software at a cross-sectional time-point. With a high acuity of psychiatric illness in the inpatient psychiatric unit, and increasing referrals, there are increased pressures for access to inpatient psychiatric beds and reduced length of stay. It is therefore plausible that patients may be discharged on antipsychotic polypharmacy with a plan to reduce and cease this following discharge.

Clozapine use is the most effective medication for treatment-resistant schizophrenia [41], and would circumvent the need for antipsychotic polytherapy for many patients. Only 11% (*n* = 43) of the total number of patients in this audit were trialled on clozapine. This low number may have been influenced by psychiatrist prescribing experiences and beliefs, underscoring the predisposition for clinicians to delay prescribing clozapine for patients with treatment-resistant schizophrenia [28]. This tendency is believed to be influenced by a range of factors, including but not limited to, clozapine’s serious adverse effect profile [42], and the perception that consumers are less satisfied with this treatment choice [43]. While this practice appears to be improving internationally [44] and in Australia [41], further investigation is needed as to the reasons for low clozapine prescribing rates in this particular hospital setting. What is known is that the increased education between Cohorts did not specifically focus on the indications and utility of clozapine. Furthermore, by sourcing clinical data from discharge summaries which are supposedly designed to be the primary clinical communication tool on discharge and transfer of care, these may not always be comprehensively authored and may therefore omit information in regards to prior clozapine trials. An underestimation of the rate of patients previously trialled on clozapine is therefore possible. It should be noted that for those patients with a previous clozapine trial, there was a greater likelihood of being prescribed antipsychotic polytherapy or polytherapy and/or high-dose. The reasons behind treatment failure with clozapine were unable to be explored in this study, but underscore the difficulty in treating patients with treatment-resistant schizophrenia especially when clozapine treatment has been stopped.

Patients diagnosed with schizophrenia in our study were also more likely to be prescribed antipsychotic polytherapy alone or with high-dose compared to those with bipolar disorder or other mental illness. The association with polytherapy alone is supported by a US based audit of outpatients visiting office-based psychiatrists [11]; other inpatient studies did not explore such associations [45], or focused solely on patients with a diagnosis of schizophrenia or related disorder [22, 24, 25]. Antipsychotics are often not first-line treatment for other mental illnesses, such as bipolar disorder. Thus, the significant relationship found between diagnosis and antipsychotic polytherapy was to some extent expected given that the majority of patients in both Cohorts had a primary diagnosis of schizophrenia.

Involuntary treatment was found to be significantly associated with antipsychotic polytherapy, with increased rates or similar associations seen in other countries [18, 46]. These vulnerable patients were also more likely to be prescribed high dose, or both polytherapy and high dose antipsychotics. Similarly, other research has confirmed that compulsory detention/involuntary patients are at a greater risk of being prescribed high antipsychotic doses [18, 24, 47, 48].

People hospitalised more than five times in the previous year were significantly more likely to be prescribed more than one antipsychotic. These consumers would have been clinically unstable; multiple antipsychotics are likely to have been prescribed as an attempt to stabilise the patient. This result underscores the need for clinicians to take particular care and attention towards both of the above vulnerable patient groups, and the reasons for such high hospitalisation rates, e.g. side effects, non-adherence, ineffective treatment, etc. must be investigated. In particular, the role of shared-decision making can be particularly important in this context; a qualitative study exploring this concept with mental health pharmacists identified the view that involving patients with serious mental illnesses in treatment decisions would improve adherence and clinical outcomes [49]. It is not known to what degree, shared-decision making was utilised in this hospital setting for audit participants as this data was not specifically captured. It is however noted that person-centred care is a core value of the service. Future audits in this area should consider the inclusion of shared-decision making as an additional variable.

Fewer variables were found to be associated with any high antipsychotic dosing compared to antipsychotic polytherapy. Of concern, however, is that overall 20% of patients were prescribed a high antipsychotic dose, placing these patients at a higher risk of adverse effects. While there are variable definitions of ‘high dose,’ the prevalence of solely high-dose prescribing in this audit was higher than that of discharged patients from a UK psychiatric ward (6.8%) [50] and Hong Kong inpatients (9.2%) [47].

An escalation protocol for identification of prescribing practices considered potentially dangerous and the need for out-of-study follow-up by an independent senior psychiatrist was included in our research design for ethics approval. In the event, no patients were identified to require use of this escalation pathway.

### Future research and practice recommendations

The observed sub-optimal antipsychotic prescribing practices at discharge from this hospital demonstrates a need for future improvement. Prescribers (training psychiatry registrars and consultant psychiatrists) need to explain the justification for treatment choices in key handover documentation such as discharge summaries. Despite the RANZCP Clinical Practice Guidelines for the Management of Schizophrenia being eight to 10 years old over the time-periods that the audit was conducted, there were deviations from recommended practice. Additionally, there remains a recommendation in the literature for antipsychotic monotherapy and the use of clozapine.

The preliminary results of the audit have influenced the service to develop a dedicated committee to explore and develop treatment pathways for psychosis. Through this committee, draft guidelines on the treatment of psychosis which further address aspects of care discussed in the audit such as antipsychotic polypharmacy, high dose antipsychotic prescription, the use of clozapine, and further augmentation strategies in treatment-resistant psychosis have been developed. These locally specific guidelines are based on current evidence and indicate a requirement to obtain consultant psychiatrist approval for high dose antipsychotic and antipsychotic polypharmacy prescription, and emphasise the need to clearly document and justify individual patient treatment decisions. The draft version of the treatment pathway for psychosis guidelines has been distributed to all training psychiatry registrars and consultant psychiatrists across the service for comment. Following finalisation of these guidelines, implementation, and a period of practice, it would be useful to analyse a third Cohort of patients to determine if there is a subsequent beneficial change to prescribing practices.

Inclusion and review of all psychotropic medications may provide a better understanding of clinical decision-making and the extent of psychotropic polypharmacy as a whole and thus total medication burden experienced by patients. Furthermore, many cases of polytherapy in our study involved combinations of oral and LAI antipsychotics; rationale for this combination regimen were not explored. Whilst this practice is not uncommon [51, 52], there is little evidence to support this practice, especially if the LAI antipsychotic was introduced to manage adherence problems with an oral medication. This is worthy of further exploration to identify patients at risk of poor treatment outcomes [51].

An aspect of psychiatric care not explored in the audit was the utilisation of pharmacists with specialist mental health expertise. This audit and the pool of literature referenced highlight the considerable variability in regards to psychiatric treatments, and the potential for negative impacts on morbidity and mortality. A future area of research may seek to compare prescribing practices in psychiatric units with dedicated mental health pharmacists and those without. If significant differences are noted, this may inform service delivery, policy creation and workforce reforms.

### Strengths and limitations

Study strengths include the standardised data collection process and large sample size. With procedural guidelines followed by all researchers, and regular debriefs, this ensured that data was collected and recorded accurately and consistently. This audit contributes to the literature by providing a better understanding of antipsychotic polytherapy and high-dose prescribing, their relationship and risk factors associated with this practice. Furthermore, this study was more inclusive by involving patients with conditions beyond schizophrenia. Finally, a key strength of this study was the exploration of variables associated with polytherapy and high dose prescribing; many published studies have focused on one or the other, not both.

However, the audit results should be interpreted within its limitations. Firstly, as a retrospective study, the quality of the data was dependent on the accuracy and completeness of clinical notes. In this particular hospital setting, clinical notes varied in terms of the quantity and quality of documented information, and there was no reliable recording of adverse effects specific to antipsychotic use. It would have been useful to consider the justification for prescribing long-term polytherapy; this information was not consistently recorded by clinicians. Unlike the work by Kadra et al. [53], the definition of antipsychotic polytherapy used did not include a time-frame; it may be that some patients were on two antipsychotics temporarily due to cross-titration, or were using an oral antipsychotic for short term use whilst commencing a long acting formulation. This information could not be identified from patient records alone in this audit; hence there is a possibility that the rates of polytherapy are over-estimated for both Cohorts [51]. With regards to additional psychotropic prescribing information, limited data regarding antidepressants, mood stabilisers and benzodiazepine medications was collected, however, it was beyond the scope of the study to widen the focus primarily because of the limited timeframes of postgraduate student research. Lastly, this audit was situated in one clinical setting in Australia, limiting the generalisability to other healthcare settings beyond this context.

## Conclusion

In a significant minority antipsychotic prescribing practices did not align with clinical guidelines, despite increased education for prescribers in-between Cohorts. This indicates that the education program alone and well established clinical practice guidelines (RANZCP) were ineffective at positively influencing antipsychotic prescribing practices. The subsequent approach to local guideline development is based on the premise of the importance of prescriber engagement in bringing about practice change. Overall, it appears that further consideration should be given when prescribing antipsychotics for involuntary patients, people with frequent hospitalisations, and those who have previously trialled clozapine. The study findings also highlight the considerable variability in psychiatric prescribing practices, and the need for further research including additional audits and evaluation of the effectiveness of formal education programs and treatment guidelines.
